# Comparison of Skull Morphometric Characteristics of Simmental and Holstein Cattle Breeds

**DOI:** 10.3390/ani14142085

**Published:** 2024-07-17

**Authors:** Buket Çakar, Faruk Tandir, Barış Can Güzel, Caner Bakıcı, Burak Ünal, Sokol Duro, Tomaz Szara, Constantin Spataru, Mihaela-Claudia Spataru, Ozan Gündemir

**Affiliations:** 1 Institute of Graduate Studies, Istanbul University-Cerrahpasa, 34320 Istanbul, Türkiye; buket.cakar@ogr.iuc.edu.tr; 2 Department of Basic Sciences of Veterinary Medicine, Veterinary Faculty, University of Sarajevo, 71000 Sarajevo, Bosnia and Herzegovina; faruk.tandir@vfs.unsa.ba; 3 Department of Anatomy, Faculty of Veterinary Medicine, Siirt University, 56100 Siirt, Türkiye; bcguzel@hotmail.com; 4 Department of Anatomy, Faculty of Veterinary Medicine, Ankara University, 06110 Ankara, Türkiye; vetcanerbakici@gmail.com; 5 Department of Anatomy, Faculty of Veterinary Medicine, Istanbul University-Cerrahpasa, 34320 Istanbul, Türkiye; burak.unal@iuc.edu.tr; 6 Department of Anatomy, Faculty of Veterinary Medicine, Agricultural University of Tirana, 1000 Tirana, Albania; durosokol@ubt.edu.al; 7 Department of Morphological Sciences, Institute of Veterinary Medicine, Warsaw University of Life Sciences-SGGW, 02-776 Warsaw, Poland; tomasz_szara@sggw.edu.pl; 8 Department of Preclinics, Faculty of Veterinary Medicine, Iasi University of Life Sciences, 700489 Iasi, Romania; 9 Department of Public Health, Faculty of Veterinary Medicine, Iasi University of Life Sciences, 700489 Iasi, Romania; mspatarufmv@yahoo.com

**Keywords:** cranium, cow, morphometry, principal component analysis, veterinary anatomy

## Abstract

**Simple Summary:**

This study examined the skull characteristics of Holstein and Simmental cattle to provide reference values for future research. We collected 54 skulls of young male cattle from Turkey to conduct 27 measurements and calculate eight indices. Holstein skulls tended to be longer, though not markedly, but they exhibited notably longer nasal bones and shorter skull lengths. Holsteins also had wider faces. A statistical analysis showed that overall size differences did not separate the breeds, but certain skull features did. Simmental skulls had higher basal index values and wider occipital regions, likely due to their larger skull size and weight, which provides more space for muscle attachment. Holsteins had more oval-shaped eye sockets, while Simmentals had wider, rounder ones. These findings help identify breed-specific traits and offer insights into how each breed’s skull structure supports their functions. This knowledge is valuable for breeding and conservation, and future research should look into the genetic and environmental factors influencing these traits.

**Abstract:**

This study aimed to reveal the morphological characteristics of pure Holstein and Simmental skulls and to obtain reference values for morphometric analysis. Moreover, 54 skulls from 12- to 14-month-old male Holstein (*n* = 25) and Simmental (*n* = 29) cattle were collected from Turkey’s Southeastern Anatolia Region between 2023 and 2024. Linear measurements indicated that Holsteins had longer skulls compared to Simmentals. Holsteins exhibited significantly higher values for the greatest length of nasals and the shortest skull length. The facial breadth was wider in Holsteins and statistically distinctive between the breeds. Holsteins had a more oval orbital bony roof, while Simmentals exhibited a wider orbital structure. The orbital index was higher in Holsteins, distinguishing between the two breeds. It was observed that Simmental cattle had a wider occipital region. This difference is likely due to the larger lateral appearance of the Simmental skull, which has more body weight and provides a larger surface area for muscle attachment. These differences not only aid in breed identification but also offer insights into the functional adaptations of each breed. Future research should explore the genetic and environmental factors contributing to these morphological traits, further enriching our knowledge of cattle morphology and its implications for breeding and conservation efforts.

## 1. Introduction

The skull is composed mainly of paired bones, forming cavities that house critical organs and systems, including the brain, the sense of smell, hearing, and balance, and the initial parts of the digestive and respiratory tracts [1]. The skull bones are connected by well-differentiated and visible sutures, which distinctly form two major parts, i.e., the cranium and the facies. The cranium, also known as the braincase, encloses and protects the brain. It consists of several bones, including the frontal, parietal, temporal, occipital, sphenoid, and ethmoid bones. These bones are intricately joined by sutures, providing rigidity and flexibility to the skull structure. The facies, or facial skeleton, supports the structures of the face and includes the bones that form the upper and lower jaws, the nasal cavity, and the orbits of the eyes. Key bones in this region include the maxilla, mandible, nasal bones, zygomatic bones, and the lacrimal and palatine bones. Understanding the detailed morphology and specific characteristics of the skull bones is essential for various fields, including veterinary anatomy, anthropology, and evolutionary biology. This knowledge allows for better diagnostic techniques, more effective treatments, and a deeper understanding of the adaptations of different species [2].

Previous studies have focused on gathering reference data for various species by examining the morphometric (measurement-based) skull features. These data have been valuable for understanding the differences in skull morphology based on sex and species. For instance, in his study, Özkan [3] compared the skulls of domestic cattle and water buffalo using linear measurements (direct measurements of the skull’s dimensions). His study identified morphometric features that distinguished the two species. Similarly, Kobryńczuk [4] studied European bison and found sex differences in their skulls using linear measurements. In another example, linear measurements were used to study the Indian Mithun, a type of cattle, and the data obtained were useful for distinguishing between sexes [5]. Research has also been conducted on sheep, dog, and cat skulls, akin to the studies performed on cattle [6,7,8,9]. These studies have been instrumental in understanding the morphological (structural) characteristics of cattle and sheep skulls and have also provided taxonomic reference values (baseline data that can be used for identifying and classifying these animals).

The Simmental is among the oldest and most widely distributed breeds of cattle in the world [10]. Historically, Simmental cattle have been used for dairy and beef production. They are particularly renowned for the rapid growth of their young, provided they receive sufficient feed. Originating in Switzerland, the breed has since spread to all six continents. Due to its high adaptability, the breed is commonly found around the world [11]. The Simmental breed, which excels in both milk and meat production, is one of the most widely distributed breeds worldwide. In Europe alone, there are about 36 million Simmental cattle, representing approximately 22% of the global population of this breed [12]. Conversely, the Holstein (Holstein, Holstein Friesian), a Dutch dairy cow breed, is known for its higher milk yield than the Simmental breed. The Holstein is the largest dairy breed in the world, boasting the highest milk production of all dairy breeds [13]. Holsteins are easily recognized by their distinctive markings, usually black and white or red and white, typically displaying piebald patterns [14]. This breed’s exceptional milk production and unique appearance have made it a staple in dairy farming worldwide.

The versatility of the Simmental breed in both meat and dairy production, combined with its adaptability to various climates, makes it an invaluable asset to the global cattle industry. Similarly, Holstein’s unparalleled milk production capabilities ensure its continued dominance in dairy farming. Together, these breeds represent the pinnacle of bovine agricultural achievement, each excelling in their respective domains. Crossbreeding studies involving these two highly productive breeds are common globally [15,16,17]. As a result of crossbreeding studies involving Holstein and Simmental cattle, the pure morphological characteristics of these two breeds may adapt over time. Although these cattle breeds are widely bred around the world and frequently used in various crossbreeding studies, research on the skull morphometry of these breeds is notably absent from reference sources. This lack of data creates a significant gap in our understanding of their morphological adaptations and genetic diversity. Studies focused on skull morphometry are essential, as they can provide insights into the evolutionary biology and functional anatomy impacts on these breeds. Furthermore, detailed morphometric analyses can aid in improving breeding programs by identifying specific traits associated with health and environmental adaptation. Therefore, addressing this gap is crucial for advancing both scientific knowledge and practical applications in cattle breeding and management. This study aims to identify and establish reference values for the morphological characteristics of Holstein and Simmental skulls. By using this reference data, skull comparisons can be made between pure breeds and hybrid races that may adapt over time, aiding in understanding the morphological changes in the skull during adaptation. In addition, the reference values obtained from this study will provide a valuable baseline for future research in veterinary morphology. These values can be used to monitor changes over generations and evaluate how selective breeding practices influence skull morphology. By establishing a comprehensive set of morphometric data, we can better understand the evolutionary trajectories of these breeds and their responses to both natural and artificial selection pressures.

## 2. Materials and Methods

### 2.1. Materials

This study utilized skulls from 12- to 14-month-old male Holstein (*n* = 25) and Simmental (*n* = 29) cattle. The average weight of Holstein and Simmental cattle skulls was 733.20 ± 65.57 kg and 1072.04 ± 101.22 kg, respectively. All samples were male and were collected from Turkey’s southeastern Anatolia Region, specifically Siirt and Diyarbakır, between 2023 and 2024. The maceration process for the skulls, collected post-slaughter, was carried out at Siirt University, Faculty of Veterinary Medicine, Department of Anatomy.

The skulls were initially cleaned by removing all soft tissues. For thorough cleaning and whitening, skulls were soaked in a 50% hydrogen peroxide solution for 1 h. This chemical treatment helps to remove any remaining organic material and ensures the bones are free of any potential contaminants. Afterward, the skulls were air-dried for 2 weeks to ensure complete residual moisture evaporation, preparing the bones fully for scanning.

### 2.2. Modeling

The prepared skulls were scanned using a Shining 3D EinScan Pro 2X (Shining 3D, Hangzhou, China) for morphometric analysis. The HD (high-definition) scanning mode was chosen to ensure detailed and accurate digital representations of the skulls. The manual scanning mode was employed to allow the operator to carefully maneuver the scanner around the skull to capture all angles and features.

The scanning speed was set at 20 frames per second, which provides a balance between capturing detail and managing the data-processing load. Additionally, the dot distance, which determines the resolution of the scan, was set to a maximum of 0.2 mm. This fine resolution ensures that even the smallest morphological features of the skulls are accurately recorded.

To avoid any interference from natural light, which can cause glare and affect the quality of the scans, the scanning was conducted in closed environments. This controlled setting ensures consistent lighting conditions, resulting in reliable and uniform scan results.

Once the scanning was completed, the raw data were processed using EXScan Pro (v4.0.0.4) software. This software is designed for mesh operations, which involves creating a 3D mesh model from the scanned data points. The mesh models are then refined and optimized for further analysis. The final models were saved in PLY (Polygon File Format), a standard format for storing three-dimensional data suitable for morphometric analysis.

### 2.3. Morphometric Analysis

All measurements were taken by the same person to ensure consistency and accuracy. The 3D Slicer image computing platform (version 5.6.2) was used to perform linear measurements in a computer environment. The process involves using 3D Slicer software to identify anatomical landmarks on the 3D models. The researcher carefully places points on these landmarks, and the software calculates the distances between them, generating precise linear measurements. This digital approach ensures high accuracy and repeatability compared to traditional manual methods.

**Figure 1:**
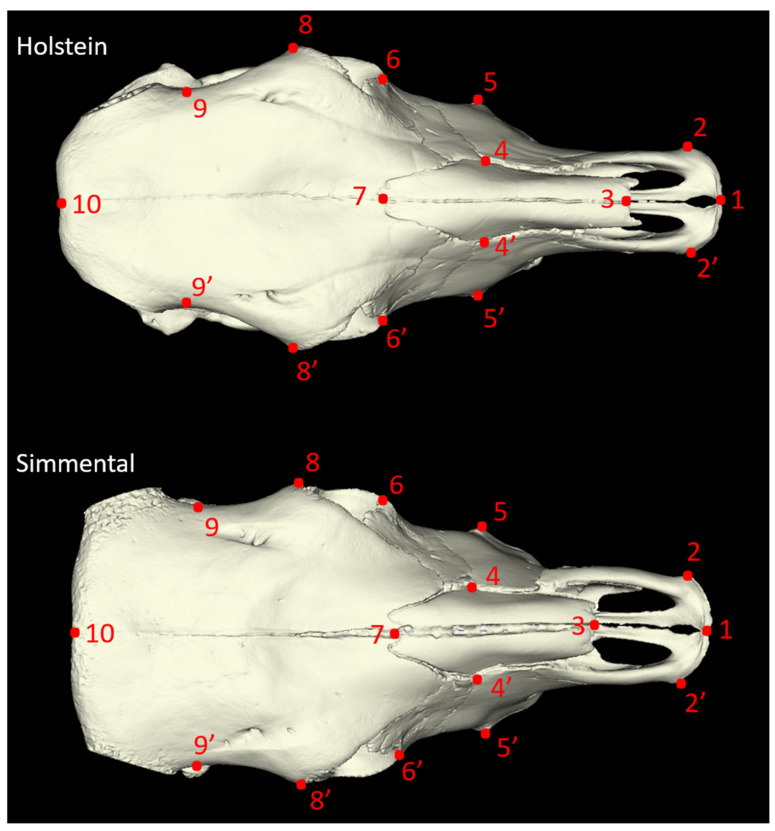
Dorsal view of the cattle skull. P1–P10: Total Length (TL): Acrocranion-Prosthion; P1–P17: Condylobasal Length (CBL): Aboral Border of the Occipital Condyles-prosthion; P7–P10: Median Frontal Length (MFL): Acrocranion-Nasion; P3–P7: Greatest Length of The Nasals (GLN): Nasion-Rhinion; P1–P8: Lateral Facial Length (LFL): Ectorbitale-Prosthion; P9–P9’: Least Frontal Breadth (LFB): Breadth of The Narrowest Part of The Frontal Aboral of The Orbits; P8–P8’: Greatest Breadth of the Skull (GBS): Ectorbitale-Ectorbitale; P6–P6’: Least Breadth Between The Orbits (LBO): entorbitale-Entorbitale; P5–P5’: Facial Breadth (FB): Across The Facial Tuberosities; P2–P2’: Breadth Across The Premaxillae on The Oral Protuberances (BPOP). The explanation for numbers for Figure 1: P1—Prosthion—Fissure Interincisivum; P2—Most Lateral Margin of Incisive Body; P3—Rostral End of Nasal Bones; P4—Nasolacrimal Fissure; P5—Facial Tuberosity; P6—Most Medial Margin of Orbits; P7—Midpoint of Nasofrontal Suture; P8—Most Caudolateral Margin of Orbits; P9—Most Medial Point of The Temporal Line; P10—Intercornual Protuberance.

**Figure 2:**
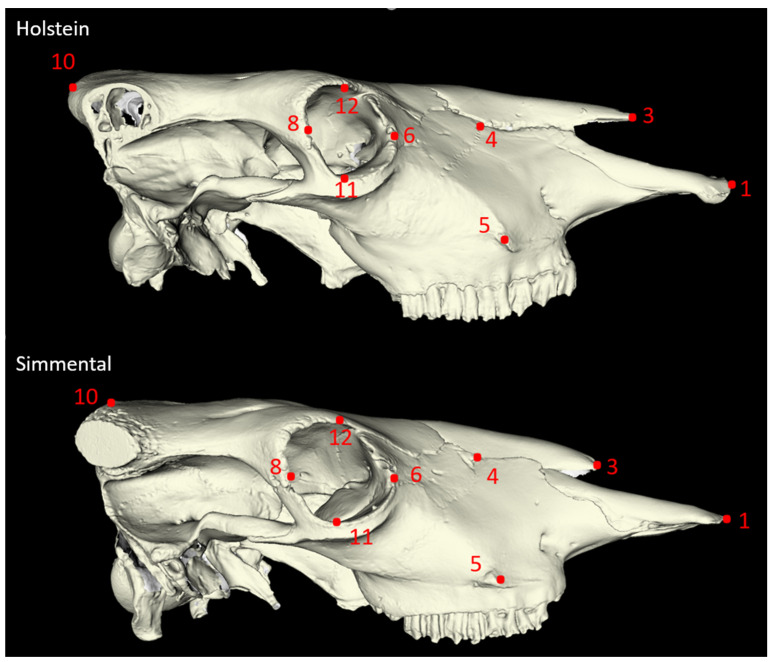
Lateral view of the cattle skull. P1–P4: Lateral Length of The Premaxilla (LLP): Nasointermaxillare-Prosthion; P6–P8: Greatest Inner Length of The Orbit (GILO): Ectorbitale-Entorbitale; P11–P12: Greatest Inner Height of The Orbit (GIHO). The explanation for numbers for Figure 2: P1—rostral margin of incisive bone; P3—rostral end of the nasal bone; P4—nasolacrimal fissure; P5—facial tuberosity; P6—most medial margin of the orbit; P8—most caudal margin of the orbit; P10—intercornual protuberance; P11—most ventral margin of orbits; P12—most dorsal margin of orbits.

**Figure 3:**
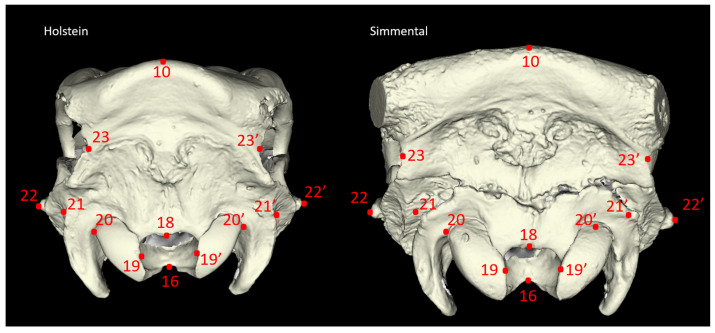
Caudal view of the cattle skull. P22–P22’: Greatest Mastoid Breadth (GMB): Otion-Otion; P20–P20’: Greatest Breadth of The Occipital Condyles (GBOC); P21–P21’: Greatest Breadth at The Bases of The Paraoccipital Processes (GBPP); P19–P19’: Greatest Breadth of The Foramen Magnum (GBFM); P16–P18: Height of The Foramen Magnum (HFM): Basion-Opisthion; P23–P23’: Least Occipital Breadth (LOB): Distance Between The Most Medial Points of The Caudal Borders of The Temporal Grooves; P10–P16: Greatest Height of The Occipital Region (GHOR): Basion-Highest Point of The Intercornual Protuberance; P10–P18: Least Height of The Occipital Region (LHOR): Opisthion-Highest Point of The Intercornual Protuberance. The explanation for numbers for Figure 3: P10—nuchal crest; P16—dorsal magin of the foramen magnum (Basion); P18—dorsal margin of foramen magnum (Opisthion); P19—latral margin of foramen magnum; P20—most lateral base of occipital condyle; P21—most lateral base of paracondylar process; P22—external acoustic meatus; P23—caudal border of temporal fossa.

**Figure 4:**
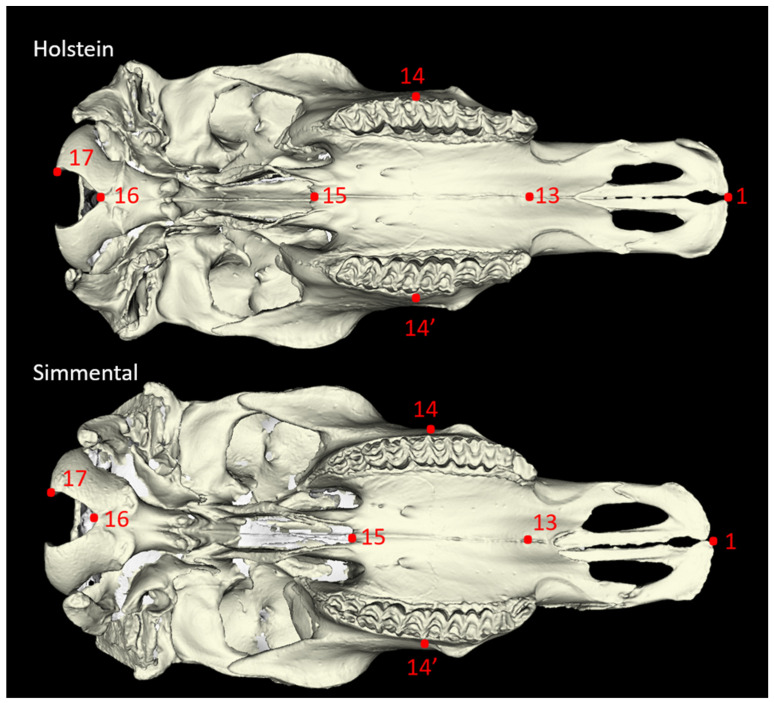
Ventral view of the cattle skull. P13–P16: Short Skull Length (SSL): Basion-Premolare; P1–P13: Premolare-Prosthion (PP); P1–P15: Dental Length (DL): Postdentale-Prosthion; P14–P14’: Greatest Palatal Breadth (GPB): Measured Across the Outer Borders of The Alveoli. The explanation for numbers for Figure 4: P1—Prosthion; P13—midpoint of the interpalatine suture at the first premolar level; P14—lateral margin of the third molar; P15—most caudal point of the interpalatine suture; P16—Basion; P17—ventromedial margin of occipital condyle.

Twenty-seven morphometric measurements were taken from each skull sample, adhering to the methodologies established by von den Driesch [18] and Özkan [3] (Figure 1, Figure 2, Figure 3 and Figure 4). In addition to these linear measurements, 8 different indexes were calculated using these measurements. Indexes are ratios or proportions derived from the measurements, providing additional insights into the skull’s shape and proportions. 

**Morphometric measurements:**

P1–P10: Total Length (Tl): Acrocranion-Prosthion

P1–P17: Condylobasal Length (Cbl): Caudal Border of The Occipital Condyles-Prosthion

P1–P16: Basal Length (Bl): Basion-Prosthion

P13–P16: Shortest Skull Length (Ssl): Basion-Premolare

P1–P13: Premolare-Prosthion (Pp)

P1–P17: Viscerocranium Length (Vcl): Nasion-Prosthion

P7–P10: Median Frontal Length (Mfl): Acrocranion-Nasion

P3–P7: Greatest Length of Nasals (Gln): Nasion-Rhinion

P1–P8: Lateral Facial Length (Lfl): Ectorbitale-Prosthion

P1–P15: Dental Length (Dl): Postdentale-Prosthion

P1–P4: Lateral Length of The Premaxilla (Llp): Nasointermaxillare-Prosthion

P6–P8: Greatest Inner Length of The Orbit (Gilo): Ectorbitale-Entorbitale

P11–P12: Greatest Inner Height of The Orbit (Giho)

P22–P22’: Greatest Mastoid Breadth (Gmb): Otion-Otion

P20–P20’: Greatest Breadth of The Occipital Condyles (Gboc)

P21–P21’: Greatest Breadth at The Bases of The Paraoccipital Processes (Gbpp)

P19–P19’: Greatest Breadth of The Foramen Magnum (Gbfm)

P16–P18: Height of The Foramen Magnum (Hfm): Basion-Opisthion

P23–P23’: Length of The Occipital Bone (Lob): Distance Between the Most Medial Points of The Caudal Borders of The Temporal Grooves

P9–P9’: Least Frontal Breadth (Lfb): Breadth of The Narrowest Part of The Temporal Line

P8–P8’: Greatest Breadth of The Skull (Gbs): Ectorbitale-Ectorbitale

P6–P6’: Least Breadth Between the Orbits (Lbo): Entorbitale-Entorbitale

P5–P5’: Facial Breadth (Fb): Across the Facial Tuberosities

P2–P2’: Breadth Across the Premaxillae on The Oral Protuberances (Bpop)

P14–P14’: Greatest Palatal Breadth (Gpb): Measured Across the Outer Borders of Dental Alveoli

P10–P16: Greatest Height of The Occipital Region (Ghor): Basion-Highest Point of The Intercornual Protuberance

P10–P18: Least Height of The Occipital Region (Lhor): Opisthion-Highest Point of The Intercornual Protuberance

**Indexes:**

Skull index: GBS/TL × 100

Facial index: FB/VCL × 100 

Frontal index: GBS/MFL × 100

Basal index: GBS/BL × 100

Length-length index: MFL/VCL × 100

Palatal index: GPB/DL × 100

Orbital index: GIHO/GILO × 100

Foramen magnum index: HFM/GBFM × 100

### 2.4. Statically Analysis

Measurements were recorded in millimeters to ensure precision and standardization. To reveal statistical differences in linear measurements and index values for distinguishing between Holstein and Simmental breeds, an analysis of variance (ANOVA) was performed. Given the unequal sample sizes, the Bonferroni method was applied to adjust for multiple comparisons and control the family-wise error rate.

Levene’s test was used to assess the homogeneity of group variances, ensuring that the required ANOVA assumptions were met. A difference was considered statistically significant if the ANOVA result yielded a *p*-value of less than 0.05 (*p* < 0.05). The Shapiro–Wilk test was used to evaluate whether the dataset conformed to a normal distribution. Results that did not show a normal distribution were stated.

Principal component analysis (PCA) was used to obtain the highest variance in all linear data. PCA is a statistical technique that transforms the dataset into a form with fewer variables while retaining the most important information. By identifying the principal components, PCA reduced the dimensionality of the data, making it easier to analyze and interpret. For the PCA, principal components that accounted for less than 10% of the total variance were not considered. This threshold ensured that only the most significant components, which capture the majority of the variability in the data, were included in the analysis.

## 3. Results

### 3.1. Analysis of Variance

Linear measurement values are provided in Table 1. TL, CBL, and BL measurements were higher in Holstein than in Simmental. However, the differences between these lengths were not statistically significant. In contrast, measurements such as GLN and SSL were significantly higher in Holstein and were found to be statistically distinctive for the breed.

Despite having a longer overall skull, the neurocranium measurements of Simmental were higher. This indicates that Simmentals have a larger neurocranium compared to Holsteins. Measurements such as LOB and LFB further supported this observation. The higher values in Simmental for these measurements were distinctive and statistically significant between the two breeds.

Facial breadth was found to be wider in Holsteins. The FB measurement in Holsteins was statistically distinctive between breeds, indicating a significant difference in the facial structure of the two breeds.

Index measurement values are provided in Table 2. Supporting the previous results, Simmental’s skull index and basal index values were higher than those in Holstein. The skull index results were not statistically significant for breed discrimination. However, the difference in the basal index was statistically distinctive between the two breeds. This finding suggests that Holsteins typically have narrower and more elongated skulls, whereas Simmentals show a broader transverse skull structure. 

The orbital index proved to be a differentiating factor between the breeds. This index value was higher in Holsteins. Specifically, the GILO value was close to the GIHO value in Holsteins, indicating a more oval-shaped orbital bony roof. In contrast, the GILO value was higher than the GIHO value in Simmentals, reflecting a rostro-caudally wider orbital structure. This morphological difference suggests that while Holsteins have a more oval orbital roof, Simmentals possess a wider orbital structure, elongated in the rostrocaudal direction.

### 3.2. Principal Component Analysis

Principal component analysis (PCA) was employed to reduce the dimensionality of the data and identify the primary components that explain the most variance. PC1 (Principal Component 1) explained 60.30% of the total variation, while PC2 (Principal Component 2) explained 12.67%.

**Figure 5:**
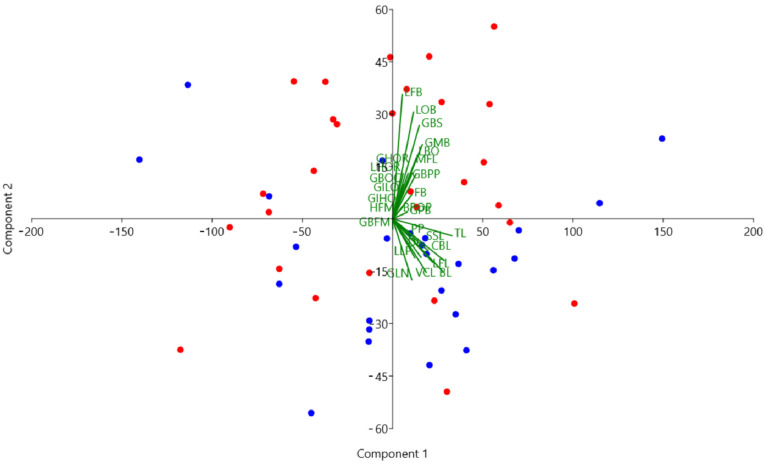
Principal component analysis scatter plot for skulls. Holstein: blue; Simmental: red.

For PC1, all linear measurements showed positive values, indicating that this component captured overall size variation among the skulls. The most effective measurement in explaining the total variance for PC1 was TL, with a PC1 value of 0.42. CBL and BL also had significant values for PC1, with PC1 values of 0.37 and 0.36, respectively. Despite the significant contributions of these measurements, PC1 did not separate the breeds, indicating that overall size was not a distinguishing factor between the Holstein and Simmental breeds (Figure 5).

For PC2, the measurements contributing to the variation were more specific. The most important measurement was LFB, with a PC2 value of 0.46. LOB and GBS also had significant values for PC2, with PC2 values of 0.39 and 0.34, respectively. Unlike PC1, PC2 statistically differentiated between the Holstein and Simmental breeds (F = 9.132, *p* = 0.0039), suggesting that these specific measurements effectively distinguished the breeds.

**Figure 6:**
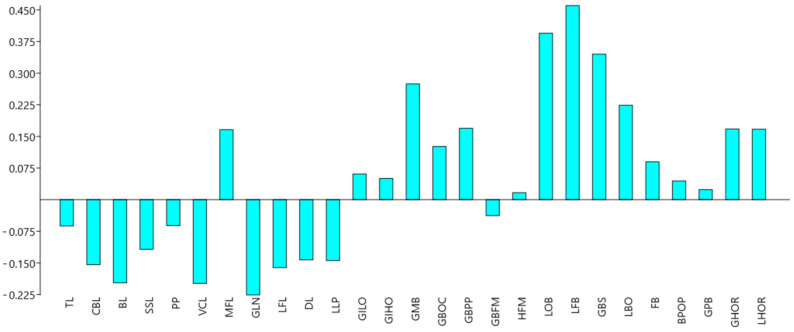
Loading plot of linear morphometric characteristics for linear measurements with the largest contributions to each dimension of the PC2 observed in skulls.

Holstein cattle exhibited a negative average PC2 value, while Simmental cattle showed a positive average PC2 value. This separation suggests that the differences in LFB, LOB, and GBS measurements were critical in differentiating the two breeds, reflecting specific morphological characteristics unique to each breed (Figure 6).

## 4. Discussion

The findings of this study provide important insights into the morphological differences between Holstein and Simmental cattle, particularly regarding skull structure. The results show that Holsteins have longer skulls overall, as indicated by measurements of TL (total length), CBL (condylobasal length), and BL (basal length). However, the lack of statistical significance in these measurements suggests that while Holsteins are generally larger, the variation within each breed may obscure clear differentiation based solely on length. Significantly higher values for GLN and SSL in Holsteins highlight distinctive morphological traits that can be used for breed discrimination. The larger neurocranium in Simmentals, supported by higher LOB and LFB measurements, suggests an adaptation that may correlate with functional or environmental factors unique to this breed. PCA further elucidates these differences by showing that while PC1 captures overall size variation, it does not effectively separate the breeds. In contrast, PC2, influenced by variations in LFB, LOB, and GBS, effectively distinguishes between Holstein and Simmental cattle. In contrast, PC2, driven by variations in LFB, LOB, and GBS, effectively distinguishes between Holstein and Simmental cattle. This separation indicates that these specific measurements are more critical for understanding breed-specific morphological adaptations. Index measurements add another layer of differentiation, with Simmentals exhibiting higher skull and basal index values, suggesting a broader skull shape. The statistically significant basal index supports the utility of this measure in distinguishing between breeds. The higher orbital index in Holsteins indicates a more oval orbital structure compared to the wider, rostrocaudal orbital roof in Simmentals.

The study results constitute essential reference values for Holstein and Simmental cattle, providing a baseline for future research and practical applications. These reference values are crucial for understanding the morphological variations within these breeds, which are widely used across the globe. For Holstein cattle, known primarily for their milk production, these values can aid in selective breeding programs aimed at enhancing productivity and health. Similarly, for Simmental cattle, which are valued for their meat yield, the reference values can inform breeding strategies to improve meat quality and overall animal health. Furthermore, these reference values are invaluable for comparative studies involving other cattle breeds. By establishing a clear morphological baseline, researchers can better identify the genetic and environmental factors that contribute to variations in skull shape and size. The novelty of this study lies in its comprehensive approach to creating these reference values using advanced 3D modeling techniques. Unlike traditional manual methods, 3D modeling ensures higher accuracy and repeatability. By pointing out specific landmarks and calculating precise linear measurements, the study offers a reliable dataset useful for a range of scientific and practical applications. This digital approach not only enhances the precision of the measurements but also allows for easier replication and verification of the results by other researchers.

The indices used in the study were derived from the pairwise ratio of various linear measurements. These indices can provide more comprehensive morphological information compared to single linear measurements. For instance, using only the TL measurement, we can determine whether the head is long or short. However, with the skull index, we can assess whether the head is thin–tall or short–wide. PCA (principal component analysis) differs from linear measurements by simultaneously analyzing all measurements and identifying those that contribute most significantly to morphological variation. While PCA considers the interrelationships among all measurements, linear analysis focuses solely on the values between two specific points. By employing both PCA and linear analysis, a more nuanced interpretation of skull morphology can be achieved, enhancing our understanding of structural variations and their underlying causes.

From linear measurements, TL was not statistically significant in distinguishing between breeds. However, in the PCA, TL was valuable in explaining the highest variance, even though it was not statistically significant for breed discrimination. PC2 values supported the results from the linear measurements that led to breed discrimination. This highlights how different data analysis methods can provide varied perspectives and insights into the results. Specifically, PCA can reveal underlying patterns in the data by transforming them into orthogonal components that explain the most variance [19]. In this study, TL, although not individually significant, played a crucial role in the first principal component (PC1), which accounted for 60.30% of the total variation. This indicates that while TL may not directly distinguish between Holstein and Simmental breeds, it is a significant factor in the overall variability of the skull measurements. On the other hand, PC2, which accounted for 12.67% of the total variation, effectively separated the breeds based on other linear measurements such as LFB and LOB, showing that these measurements were more influential in distinguishing between Holstein and Simmental. The combination of PCA and linear measurements thus provided a more comprehensive understanding of the morphological differences between the breeds [20,21]. This underscores the importance of using multiple analytical approaches to evaluate complex biological data. Different methods can uncover various aspects of the data, leading to a more nuanced and thorough interpretation of the results. By integrating PCA with traditional linear measurements, researchers can gain deeper insights into the morphological characteristics and potential adaptations of different cattle breeds.

Foramen magnum linear measurements have been extensively utilized in various animals, leading to significant morphometric conclusions. For instance, Özkan [3] reported that the height of the HFM is a distinguishing feature between cattle and water buffalo. Similarly, Akbaş [22] found this measurement to be a decisive factor between sexes in his studies on cats. Kupczyńska et al. [23] examined the foramen magnum in different dog breeds and identified four distinct structures, i.e., oval, pentagonal, rhomboid, and circular. In our study of Holsteins and Simmentals, the foramen magnum measurements were very close to each other and did not show significant differences. This suggests that while foramen magnum measurements may not distinguish Holstein and Simmental cattle, they could be useful discriminatory features for other breeds in future research.

Holstein had a lower skull index (43.64) compared to Simmental (44.87), indicating that Holsteins have a relatively longer skull compared to their width than Simmentals. According to Özkan’s study, water buffalo have a skull index of 43.01, indicating a relatively longer and narrower skull than Holsteins and Simmentals.

Also, in the same study (ref. [3]), the skull of an unknown breed, with a skull index of 43.23, falls between the skull indices of Holsteins and Simmentals. Holsteins, with their larger size and longer skull dimensions, exhibit a more elongated skull than Simmentals, which have a relatively broader skull shape. Water buffalo (TL: 471.97; GBS: 202.80), known for their robust build, have a skull shape that reflects their sturdy nature, with a relatively shorter and narrower skull compared to both Holsteins and Simmentals. The specimens discussed in Özkan’s article, though unspecified, exhibit morphology more similar to that of Holsteins, with a relatively elongated skull. These findings offer insights into the skull morphologies of these species and carry significant taxonomic implications.

When comparing the skull indices of sheep and cattle, it becomes evident that sheep exhibit a longer and narrower skull shape in contrast to the generally wider skull of cattle [6,7]. This disparity in skull morphology is likely influenced by the wider structure of the frontal bone in larger ruminants, such as cattle, which contributes to their broader skull shape. This wider frontal bone provides ample space for the attachment of muscles involved in chewing and head movements, which are more pronounced in larger-bodied animals like cattle. Furthermore, the similarity in orbit index values between certain sheep breeds and Simmental cattle indicates a potential overlap in orbital characteristics among these breeds. For instance, the orbital index values for Simmental cattle (92.86), Bardhoka sheep (93.06), and Sharri sheep (92.26) show orbital features close to each other [6,7]. Conversely, the rounder orbit index observed in Holstein cattle (98.27) compared to other ruminants may be attributed to breed-specific adaptations or genetic factors unique to Holsteins.

The occipital region of the skull plays a crucial role in head movement, housing important muscles such as those involved in neck extension and flexion. The nuchal ligament, attaching to the external occipital protuberance, provides support and stability to the head during movement. In larger ruminants, like cattle, the occipital region is particularly significant due to the substantial weight of the skull and the need for efficient head movements for feeding and other activities. Higher basal occipital area measurements (GBPP, LOB, GMB, and GBOC) in Simmental cattle than in Holstein cattle indicate a larger occipital region in Simmental skulls. This difference is likely due to the broader lateral aspect of the Simmental skull, which provides a larger surface area for muscle attachment. The increased size of the occipital region in Simmental cattle is predictable given their larger average weight compared to Holsteins. These findings underscore the importance of considering skull morphology in light of breed-specific adaptations and functional requirements. The differences in basal occipital area measurements between Simmental and Holstein cattle highlight the structural variations shaping their respective skull forms and overall biomechanics.

While each linear measurement may not provide a complete picture of the overall head morphology, it can offer valuable insights into the specific bones from which the measurement is taken. For instance, in our study, GLN was significantly greater in Holstein samples compared to Simmental samples, indicating a distinct difference in nasal bone length between the two breeds. These reference data are crucial in archeology, where researchers often rely on animal bone samples obtained during excavations to identify species and understand the taxonomy of past fauna. Current linear reference data serve as a valuable tool for these purposes. When archeologists find isolated bones, they can compare these measurements to established reference data to make educated guesses about the species, breed, or even the period the animal might have lived in [24,25]. For example, a nasal bone sample with measurements similar to the GLN values for Holstein cattle can be tentatively identified as belonging to a Holstein. However, it is important to acknowledge that reference data from only two animal samples is insufficient for broad taxonomic conclusions. To develop robust and reliable reference datasets, researchers need to conduct extensive studies involving more cattle samples from various breeds. By accumulating and analyzing more data, we can enhance the accuracy and comprehensiveness of linear reference datasets, thus enriching the taxonomic bibliography and providing more precise tools for species identification in archeological contexts [26]. However, applying these findings directly to archeological contexts presents several potential challenges. One major issue is taphonomic changes, which refer to the various processes that affect animal remains from the time of death to their discovery. These processes, including weathering, soil acidity, and pressure from overlying sediments, can alter the bones’ morphology and complicate direct comparisons with modern reference data. Additionally, the original context of the bones, such as their burial conditions and potential for distortion or damage over time, must be carefully considered. Therefore, while the study provides valuable reference data for Holstein and Simmental cattle, caution is needed when interpreting these findings in archeological settings, as post mortem changes could significantly impact the accuracy and reliability of morphological assessments.

Traditional manual measurement techniques are often subject to inconsistencies due to the variability in human perception and physical limitations. In contrast, 3D modeling allows for consistent, repeatable measurements, which are crucial for scientific studies requiring high precision. In summary, the use of 3D models in morphometric studies represents a significant advancement in the field. It provides an unparalleled level of accuracy and repeatability, ensuring that researchers can obtain reliable data. This technological innovation not only improves the quality of current research but also sets a new standard for future studies in anatomical and morphometric analysis.

## 5. Conclusions

Potential studies on the environmental factors influencing skull shape development could focus on investigating how specific climatic conditions, dietary patterns, and habitat types contribute to morphological adaptations in cattle skulls. For example, comparative analyses across different geographical regions could elucidate how temperature variations influence skull size and shape, particularly in breeds adapted to extreme climates. Longitudinal studies tracking skull development in cattle raised under controlled environmental conditions versus natural habitats could provide insights into adaptive responses over generations. In conclusion, this study on Holstein and Simmental cattle skull morphology not only establishes valuable reference values but also underscores the complex interplay between genetic and environmental factors shaping skull characteristics. By employing advanced methodologies like PCA and morphometric indices, this research contributes significantly to our understanding of how breed-specific traits are influenced by both biological and environmental variables. These findings not only enhance our knowledge of cattle adaptation and evolution but also pave the way for future research exploring broader implications in evolutionary biology and animal breeding practices.

## Figures and Tables

**Table 1:** The results of the linear measurements (ANOVA).

Measurement	Holstein	Simmental	Minimum	Maksimum	F Statistic	*p*-Value
	Mean ± SD	Mean ± SD				
TL	490.59 ± 28.08	485.97 ± 24.3	427	553.7	0.4208	0.5194
CBL	475.62 ± 25.06	467.39 ± 19.31	412.8	520.4	1.8537	0.1792
BL	445.22 ± 24.43	435.07 ± 20.34	382.2	490.6	2.778	0.1016
SSL	315.96 ± 17.59	303.53 ± 14.84	268.5	346.5	7.9235	**0.0069**
PP	129.08 ± 9.60	131.46 ± 8.84	101.1	152.6	0.8916	0.3494
VCL	267.62 ± 17.58	263.03 ± 17.27	221.4	303.4	0.9336	0.3384
MFL	223.21 ± 15.93	226.51 ± 13.82	187.8	261.4	0.6627	0.4193
GLN	172.08 ± 14.60	163.22 ± 17.13	130.1	198.1	4.1133	**0.0477**
LFL	332.52 ± 29.40 *	335.46 ± 15.56	216.6	370.6	0.2185	0.6421
DL	265.51 ± 14.60	260.04 ± 12.51	231.3	295.5	2.1946	0.1445
LLP	150.27 ± 17.70	146.84 ± 10.61	112.1	175.3	0.77	0.3842
GILO	70.17 ± 4.53	72.82 ± 5.20 *	59.4	78.86	3.9193	0.05304
GIHO	68.84 ± 5.13	67.50 ± 4.78	58.24	80.2	0.9968	0.3227
GMB	210.98 ± 16.95	212.20 ± 15.28	178.3	256	0.07782	0.7814
GBOC	109.71 ± 7.31	112.78 ± 7.54	94.85	128	2.1978	0.1442
GBPP	156.64 ± 11.66	159.59 ± 11.01	130.4	179.3	0.9101	0.3445
GBFM	40.10 ± 4.48 *	40.41 ± 5.85	28.13	52.68	0.05247	0.8197
HFM	39.35 ± 3.18 *	38.98 ± 4.35	29.4	47.25	0.1246	0.7255
LOB	137.42 ± 12.20	146.60 ± 16.28	111.1	178.2	5.356	**0.0246**
LFB	164.52 ± 10.08	175.74 ± 15.64	142.9	201	9.4663	**0.0033**
GBS	213.96 ± 15.09	218.02 ± 15.44	182.8	249.9	0.9498	0.3343
LBO	166.44 ± 13.47	169.60 ± 13.50	138.8	206	0.7368	0.3946
FB	160.56 ± 11.49	153.71 ± 10.45	129.4	195.4	5.2528	**0.0259**
BPOP	82.49 ± 9.19 *	83.79 ± 4.92	47.17	94.86	0.4415	0.5093
GPB	138.97 ± 8.45	135.26 ± 7.51	116.7	164.1	2.9181	0.0935
GHOR	158.63 ± 9.32	157.64 ± 12.45	132.6	179.9	0.1065	0.7455
LHOR	122.16 ± 9.60	122.02 ± 10.75	103.8	140.9	0.002455	0.9607

* Measurements that do not show a normal distribution according to the Shapiro–Wilk test. **Bold:** The differences between breeds are statistically significant.

**Table 2:** The results of the indexes (ANOVA).

Measurement	Holstein	Simmental	F Statistic	*p*-Value
	Mean ± SD	Mean ± SD		
Skull index	43.64 ± 2.45	44.87 ± 2.43	3.3919	0.07122
Facial index	60.09 ± 3.67	58.62 ± 4.85	1.5412	0.2200
Frontal index	96.03 ± 5.93	96.40 ± 6.43	0.0467	0.8298
Basal index	48.09 ± 2.78	50.13 ± 3.01	6.6093	**0.0131**
Length-length index	83.56 ± 6.06	86.38 ± 7.69	2.1873	0.1452
Palatal index	52.37 ± 2.07	52.06 ± 2.74	0.2063	0.6516
Orbital index	98.27 ± 6.80	92.86 ± 5.46	10.5238	**0.0020**
Foramen magnum index	99.32 ± 13.58	98.21 ± 16.83	0.06908	0.7937

**Bold:** The differences between breeds are statistically significant.

## Data Availability

The data presented in this study are available upon request from the corresponding author.
